# Brief Research Report: Quantitative Analysis of Potential Coronary Microvascular Disease in Suspected Long-COVID Syndrome

**DOI:** 10.3389/fcvm.2022.877416

**Published:** 2022-05-31

**Authors:** Patrick Doeblin, Fridolin Steinbeis, Cian M. Scannell, Collin Goetze, Sarah Al-Tabatabaee, Jennifer Erley, Alessandro Faragli, Felix Pröpper, Martin Witzenrath, Thomas Zoller, Christian Stehning, Holger Gerhardt, Javier Sánchez-González, Ebraham Alskaf, Titus Kühne, Burkert Pieske, Carsten Tschöpe, Amedeo Chiribiri, Sebastian Kelle

**Affiliations:** ^1^Department of Internal Medicine and Cardiology, German Heart Center Berlin, Berlin, Germany; ^2^German Centre for Cardiovascular Research (DZHK), Berlin, Germany; ^3^Department of Infectious Diseases and Respiratory Medicine, Charité – Universitätsmedizin Berlin, Corporate Member of Freie Universität Berlin, Humboldt-Universität zu Berlin, Berlin, Germany; ^4^School of Biomedical Engineering and Imaging Sciences, King’s College London, London, United Kingdom; ^5^Swiss Tropical and Public Health Institute, Basel, Switzerland; ^6^Clinical Science, Philips Healthcare, Hamburg, Germany; ^7^Integrative Vascular Biology Laboratory, Max-Delbrück Center for Molecular Medicine in the Helmholtz Association (MDC), Berlin, Germany; ^8^Philips Healthcare, Madrid, Spain; ^9^Department of Congenital Heart Disease, German Heart Center Berlin, Berlin, Germany; ^10^Department of Cardiology, Campus Virchow Klinikum, Charité – Universitätsmedizin Berlin, Corporate Member of Freie Universität Berlin, Humboldt-Universität zu Berlin, Berlin, Germany; ^11^BIH Center for Regenerative Therapies (BCRT), Berlin Institute of Health at Charite (BIH), Universitätsmedizin Berlin, Berlin, Germany

**Keywords:** CMR, COVID-19, microvascular disease, quantitative perfusion, long COVID-19 syndrome

## Abstract

**Background:**

Case series have reported persistent cardiopulmonary symptoms, often termed long-COVID or post-COVID syndrome, in more than half of patients recovering from Coronavirus Disease 19 (COVID-19). Recently, alterations in microvascular perfusion have been proposed as a possible pathomechanism in long-COVID syndrome. We examined whether microvascular perfusion, measured by quantitative stress perfusion cardiac magnetic resonance (CMR), is impaired in patients with persistent cardiac symptoms post-COVID-19.

**Methods:**

Our population consisted of 33 patients post-COVID-19 examined in Berlin and London, 11 (33%) of which complained of persistent chest pain and 13 (39%) of dyspnea. The scan protocol included standard cardiac imaging and dual-sequence quantitative stress perfusion. Standard parameters were compared to 17 healthy controls from our institution. Quantitative perfusion was compared to published values of healthy controls.

**Results:**

The stress myocardial blood flow (MBF) was significantly lower [31.8 ± 5.1 vs. 37.8 ± 6.0 (μl/g/beat), *P* < 0.001] and the T2 relaxation time was significantly higher (46.2 ± 3.6 vs. 42.7 ± 2.8 ms, *P* = 0.002) post-COVID-19 compared to healthy controls. Stress MBF and T1 and T2 relaxation times were not correlated to the COVID-19 severity (Spearman *r* = −0.302, −0.070, and −0.297, respectively) or the presence of symptoms. The stress MBF showed a U-shaped relation to time from PCR to CMR, no correlation to T1 relaxation time, and a negative correlation to T2 relaxation time (Pearson *r* = −0.446, *P* = 0.029).

**Conclusion:**

While we found a significantly reduced microvascular perfusion post-COVID-19 compared to healthy controls, this reduction was not related to symptoms or COVID-19 severity.

## Introduction

Case series have reported persistent cardiopulmonary symptoms, often termed long-COVID or post-COVID syndrome, in more than half of patients recovering from COVID-19 (1–3). The underlying pathology may include myocardial and endothelial abnormalities due to the virus or activation of the immune and coagulation systems. Several cardiac magnetic resonance (CMR) imaging studies have found alterations in functional and tissue parameters without relation to symptoms (4). Recently, alterations in microvascular perfusion have been proposed as a possible pathomechanism in long-COVID syndrome (5).

We examined whether microvascular perfusion, measured by quantitative stress perfusion CMR, is impaired in patients with persistent cardiac symptoms post-COVID-19.

## Method

Our population consisted of 33 patients post-COVID-19 examined in Berlin and London, 11 (33%) of which complained of persistent chest pain and 13 (39%) of dyspnea. The study was approved by the Local Ethics Committees (DRKS 00021688). The COVID-19 severity was assessed according to the WHO COVID-19 Clinical Management Guidance (6). The data from 17 healthy controls for T1 and T2 relaxation times were taken from unpublished data from our institution (DRKS 00013253). The stress agent used was adenosine in 13 and regadenoson in 20 patients. To adjust for the higher stress heart rate under regadenoson compared to adenosine, the myocardial blood flow (MBF) per heartbeat was calculated. For stress MBF per heartbeat, no normal values exist for the 3.0T Philips scanners. The published values for a 1.5T Siemens scanner were used (7).

**FIGURE 1:**
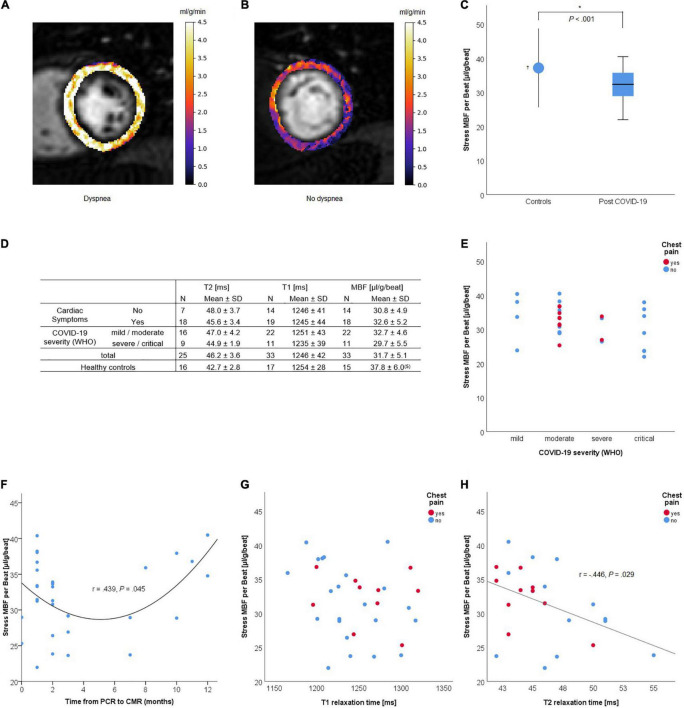
Quantitative dual-sequence myocardial stress perfusion in patients who recovered from COVID-19. **(A,B)** Examples of quantitative perfusion maps of stress myocardial blood flow (MBF) (ml/min/g) before heart rate correction for a patient with high [**(A)**, median 4.1 ml/g/min]; and low [**(B)**, median 1.8 ml/g/min] perfusion values. **(C)** Stress MBF (μl/g/beat) in post-COVID-patients compared to healthy controls. **(D)** Quantitative MRI parameters by symptoms and COVID-19 severity. **(E–H)** Stress MBF by initial COVID-19 severity **(E)**, time from PCR to CMR, T1 **(G)** and T2 **(H)** relaxation times (ms) and the presence of chest pain at the time of cardiac magnetic resonance (CMR) imaging. ^†^Data from Vasu et al. (7). *Statistically significant (*P* < 0.05).

All scans of patients were performed on the Philips Ingenia 3.0T scanners according to recent recommendations for CMR in patients post-COVID-19 (8). The scan protocol included standard cardiac imaging and dual-sequence quantitative stress perfusion (9, 10). Exemplary MBF maps of patients with high and low MBFs are shown in Figures 1A,B, respectively.

## Results

The median age of the 33 post-COVID-19 patients was 51 years (interquartile range, IQR 42–58) with 52% being women. The symptoms at time of CMR were chest pain (33.3%), dyspnea (39.4%), fatigue (42.4%), and arrhythmia (6.1%), with any of the above in 57.6% of the patients. The median age of the 17 healthy controls for T1 and T2 relaxation times was 24 years (IQR 21–25) with 50% being women. The median time between the first positive PCR for COVID-19 and the CMR exam was 2 months (IQR 1–5).

One patient was excluded from MBF per heartbeat analysis due to atrial fibrillation with varying heart rates. T2 relaxation times were available for 25 patients. The heart-rate adjusted stress MBF was significantly lower [31.8 ± 5.1 vs. 37.8 ± 6.0 (μl/g/beat), *P* < 0.001] and the T2 relaxation time was significantly higher (46.2 ± 3.6 vs. 42.7 ± 2.8 ms, *P* = 0.002) post-COVID-19 compared to healthy controls (Figures 1C,D). Stress MBF and T1 and T2 relaxation times were not correlated to age (*r* = 0.233, 0.040, and 0.009, respectively), COVID-19 severity (Spearman *r* = −0.302, −0.070, and −0.297, respectively), or the presence of symptoms (Figures 1D,E). The stress MBF showed a U-shaped relation to time from PCR to MRI, no correlation to T1 relaxation time, and a negative correlation to T2 relaxation time (Pearson *r* = −0.446, *P* = 0.029) (Figures 1F–H).

One patient with a history of myocarditis showed non-ischemic late gadolinium enhancement (LGE) consistent with subsided myocarditis. No patient showed myocardial edema and no patient fulfilled the updated Lake Louise criteria for acute myocarditis based on the CMR examination (11). One patient had focal pericarditis. Five patients had a stress-induced regional perfusion deficit, four of which were consistent with coronary artery disease and one with microvascular dysfunction in the presence of left ventricular hypertrophy. Excluding the five patients with regional ischemia did not significantly alter the mean stress MBF [32.7 ± 5.0 (μl/g/beat), *P* = 0.005 vs. controls] or T2 relaxation time (45.7 ± 2.8 ms, *P* = 0.004 vs. controls). Interestingly, the stress MBF was not correlated to clinical symptoms or severity of COVID-19.

## Discussion

A recent study evaluated the relationship between symptoms and functional alterations in hospitalized COVID-19 patients. The findings demonstrated that while 10 months after discharge from a hospital stay due to COVID-19 the percentages of patients with symptoms were high, those symptoms could not be attributed to altered lung function or physical capacity (12).

In conclusion, while we found reduced microvascular perfusion post-COVID-19 compared to external, non-age-matched healthy controls, this reduction was not related to symptoms and COVID-19 severity. However, a potential temporary decrease in coronary microvascular perfusion might be linked to potentially affected endothelial function also in other organ regions and therefore might play a role in the symptoms described by the patients months after COVID-19.

## Limitations

A significant limitation is the use of external normal values for stress MBF per heartbeat from a single study of 15 healthy volunteers with a median age of 21 years, scanned on a scanner of different field strengths from a different vendor. While a confounding effect of age and comorbidities is possible, stress MBF per heartbeat was not related to age or the presence of a regional perfusion deficit in our dataset.

Further studies, including age-adjusted normal values and serial measurements of patients with hampered MBF in relation to clinical findings, are needed to better define the long-COVID syndrome and establish a causal relationship to reduced MBF.

## Data Availability Statement

The raw data supporting the conclusions of this article will be made available by the authors, without undue reservation.

## Ethics Statement

The studies involving human participants were reviewed and approved by the Ethikkommission der Charité Universitätsmedizin Berlin. Written informed consent for participation was obtained as required in accordance with the national legislation and the institutional requirements.

## Author Contributions

All authors listed have made a substantial, direct, and intellectual contribution to the work, and approved it for publication.

## Conflict of Interest

PD owns stock of Siemens and Bayer. AF was a shareholder of BOCAhealthcare GmbH. CS was an employer of Philips Healthcare. BP reported receiving personal fees from Bayer, Bristol Myers Squib, Daiichi Sankyo, Medscape, MSD, Novartis, Stealth Peptides, and Vifor Pharma, and grants and personal fees from Astra-Zeneca. CS and JS-G were employees of Philips Healthcare. CT, BP, and SK received funding from the DZHK (German Centre for Cardiovascular Research) and by the BMBF (German Ministry of Education and Research) and personal fees from Servier, outside of the current work. SK received an unrestricted research grant from Philips Healthcare and received lecture honoraria from Medis, NL. The remaining authors declare that the research was conducted in the absence of any commercial or financial relationships that could be construed as a potential conflict of interest.

## Publisher’s Note

All claims expressed in this article are solely those of the authors and do not necessarily represent those of their affiliated organizations, or those of the publisher, the editors and the reviewers. Any product that may be evaluated in this article, or claim that may be made by its manufacturer, is not guaranteed or endorsed by the publisher.
